# Assessment of spatial autocorrelation and scalability in fine-scale wildfire random forest prediction models

**DOI:** 10.1038/s41598-025-06814-z

**Published:** 2025-07-01

**Authors:** Madeleine Pascolini-Campbell, Joshua B. Fisher, Kerry Cawse-Nicholson, Christine M. Lee, Natasha Stavros

**Affiliations:** 1 NASA Jet Propulsion Laboratory, California Institute of Technology, Pasadena, CA USA; 2 Chapman University, Orange, CA USA; 3 WKID Solutions LLC, Boulder, CO USA

**Keywords:** Fire ecology, Forest ecology, Forestry, Natural hazards

## Abstract

Wildfire prediction models that can be applied across diverse regions at fine scales (< 100 m) are critical for wildfire management. Remote sensing offers a path forward by providing heterogeneous and dynamic measurements of fuel load, type, and flammability. Machine learning methods such as random forests provide an empirical framework that are high-accuracy, computationally efficient, interpretable and able to model complex ecological relationships. Here we use high resolution (70 m, every 3–5 days) remote sensing observations of evapotranspiration and evaporative stress index, which represent plant water stress, from Ecosystem Spaceborne Thermal Radiometer on Space Station (ECOSTRESS), as well as topography and weather data, to predict burn severity and occurrence for 8 large wildfires that burned 3715 km^2^ from 2021 and 2022 in New Mexico, USA. These fires ranged from low to high burn intensity, and covered a diverse range of ecoregions (deserts, grasslands, forests), plant species, and topographies. We used a single model to predict the burn severity of all wildfires one week before occurrence. The prediction accuracy was greatest when using all predictors (ECOSTRESS, weather, topography) (R^2^ = 0.77). We assessed the role of spatial autocorrelation in driving model performance by: (1) increasing the sample spacing of our dataset, (2) introducing new predictors that represent spatial structure in the data, and (3) training our model on half the fires and predicting the other half of the fires. We found that after increasing sample spacing, model accuracy declined. However, we found declines in model accuracy were more impacted by decreased training set size compared to the distance spacing-indicating that the models are likely accurately capturing fine-scale processes. Scalability of random forest models was also found to be more challenging for regression problems but was accurate for classification of burned pixel occurrence (total pixel accuracy of 67%). These results provide promising results for application of random forest models to predict fine-scale fire severity and occurrence with applications for fire management.

**Supplementary Information:**

The online version contains supplementary material available at 10.1038/s41598-025-06814-z.

## Introduction

Wildfires in the Western United States (US) are increasing in frequency and severity^1–3^, with associated impacts on society^4^, ecosystems^5^, hydrology^6^, and the carbon cycle^7^. Wildfires occur across diverse landscapes and burn through different vegetation types and at different severities. Low-severity fire can provide many ecological benefits in Western US ecosystems where fire is a natural environmental process. Large high-severity fires create potential for ecosystem transitions^5^, loss of carbon storage^7^, and potential for post-fire hazard such as debris flows, water quality impacts and stream sedimentation^6^. Predicting where the most severe impacts of wildfire and highest wildfire risk may occur at regional scales and in diverse landscapes, as well as regions suited to low-severity beneficial fires, is essential for pre-fire fuels mitigation.

Burn severity – a metric of the damage to soils and vegetation from wildfire – is highly heterogeneous at fine-scales and depends on both weather and antecedent fuels and their flammability^8^, as well as land cover and topography^9^. Burn severity can be measured from remote sensing using the ‘differenced normalized burn ratio’ (dNBR) method by comparing near and mid infrared reflectance from before and after the fire^10^. Near infrared is reflected by healthy vegetation, while mid infrared is largely reflected by bare soil and rock and can therefore be used to inform changes to landscapes post-fire. Different classes describe damage post wildfire, with ‘low severity’ describing light impacts to canopy, and burning of surface litters, ‘moderate severity’ describing burning of the understory plants and soils, damage to the canopy, and ‘high severity’ including canopy tree mortality and understory consumed by fire^11^^,^^12^. These classes can be related to satellite-based dNBR metrics and though uncertainty exists, have been found to match field surveys^13^. In this study we use the breakpoints based on dNBR set by the United States Geological Survey to quantitatively represent the above descriptors^14^. dNBR from Burned Area Emergency Response (BAER) teams provides an assessment from within 7 days of fire containment, although assessments can also take place before the fire is fully contained. This dataset primarily captures damage done to soils but also captures vegetation damage and can inform ensuing environmental hazards to do with fire including soil erosion, flooding, landslides, and ecosystem regeneration^12^. The information provided by BAER assessments is critical for management teams to prepare emergency response and rehabilitation plans, to drive hazard models for downstream impacts, and to understand ensuing impacts on vegetation rehabilitation (see https://burnseverity.cr.usgs.gov/baer/).

Both physics-based and empirical models have been employed to model wildfire effects and behavior. Physics-based models solve equations that describe the physical processes influencing different fire behaviors and are useful for simulating multiple fire parameters of interest which are not easily observed. In the present study we choose an empirical approach, given the advantage of producing fire predictions over large geographic areas which is the goal of this study. There has been a proliferation of empirical-based modeling of wildfires using machine learning aimed at predicting wildfire occurrence^15–18^, and burn severity^19–23^. Machine learning models have the advantage of resolving complex relationships without physics-based rules or data inputs which may be lacking to drive physics-models. Random forests have emerged as among the most popular in wildfire prediction given that they have high accuracy, are computationally efficient^24^, and are effective at resolving complex non-linear relationships between ecological and climatological variables (for example, the complex behaviors of fuels-topography-weather)^20^. Random forest model results are also interpretable through the assessment of feature importance which is useful for understanding the underlying processes driving model performance^24^. Random forests are a type of supervised machine learning algorithm, which model labeled target data which can be either continuous (regression) or categorical (classification)^25^.

Burn severity patterns have been predicted at fine-scales (< 100 m) using random forest models, and these studies have indicated the importance of including information on both fuel and weather factors, as well as landscape factors (topography and vegetation type)^21,22,26,27^. These studies are valuable for understanding the contributions of diverse drivers of burn severity at fine-scales, however they have often focused on individual fires^19^, or regions with similar characteristics^21,22^. An advantage of machine learning based approaches to wildfire hazard prediction is that they can be easily implemented to different regions, and do not need to be spatially calibrated for certain areas^24^. They also can handle complex relationships existing between predictor variables, without requiring physics-based rules^24^. In recent years, the amount of data (from both remote sensing and in situ networks) measuring fire-relevant variables has also proliferated, providing vast data sets for empirical fire modeling^24^. This all suggests the utility of such methods in modeling wildland fire behavior across diverse areas.

Empirical studies have been used to produce regional fire forecasts using satellite remote sensing data. For example, Farahmand et al.^28,29^ used a logistic regression modeling framework with vapor pressure deficit (VPD) from the Atmospheric Infrared Sounder (AIRS) instrument, and water storage information from the Gravity Recovery and Climate Experiment (GRACE) satellites, to produce binary predictions of wildfire occurrence for the continental US (0.25 degrees resolution). Another study also focused on producing binary predictions of wildfire occurrence for the Western US, training the models on historical patterns of temperature and moisture deficit^30,31^ predicted wildfire risk in California using both natural and human predictors at a scale of 1 km. These fire outlooks cover large regions but are binary in nature (indicating whether the model predicts a pixel burned or did not burn) which can be less instructive than burn severity predictions which give outlooks on expected damage to vegetation and soils. Predicting expected severity of burns can be important for assessing the potential hazard, for managers planning prescribed burns who might be interested in producing low-severity fires or allowing natural wildfires to burn at low severity, as well as for informing post-fire impacts including debris flow risks. Having high spatial resolution (at the scale of an individual stand of trees ~ 100 m or less), is important for these activities.

Here we produce a wildfire prediction model for the state of New Mexico using satellite remote sensing predictors with a lead-time of one week. We use ECOsystem Spaceborne Thermal Radiometer on Space Station (ECOSTRESS) data to characterize vegetation fuel amount and water stress (flammability). ECOSTRESS data provides high spatial (70 m) and temporal (3 to 5 day) resolution data for different metrics of plant water stress: evapotranspiration (ET) and evaporative stress index (ESI)^32^. ET—which is the sum of soil evaporation and transpiration from plants—indicates where plants are transpiring and represents vegetation accumulations and has been found to be positively related to burn severity^22,23,26^. ESI—the climatological ratio of potential ET to actual ET^33^- represents water stress in plants and can be considered a proxy for vegetation flammability^34^. These variables uniquely provide information at fine-scales appropriate for the complex landscapes impacted by wildfires^22,35^.

Spatial autocorrelation – or the degree of similarity of neighboring pixels – also plays a large role in these types of ecological models due to the relatedness of adjacent environmental conditions^36^. Given that burn severity and model predictors (plant water stress, topography, weather) co-vary together along environmental gradients, burn severity patterns will retain the spatial patterns of the input ecological variables^20^. For example, it is expected that neighboring pixels will have similar vegetation and topographical characteristics, resulting in the variables being autocorrelated in space and related values of dNBR. Patterns of dNBR themselves are expected to have spatial relatedness due to microclimates or conditions that drove burning rates^19^. Accounting for spatial autocorrelation is needed to assess whether model accuracy is driven by the model accurately capturing processes or driven by inherent spatial autocorrelation. Not accounting for spatial autocorrelation can result in the model simply matching nearby similar conditions (model overfitting) which could reduce scalability of the ecological model to other regions^19^. Kane et al.^19^ showed that increasing the sample spacing of their model input data reduced the spatial autocorrelation but maintained consistent relationships between predictor variables (from feature importance). The Principal Coordinates of Neighbor Matrices (PCNM) method can also be applied, which introduces new predictors that represent spatial structure of the predictor data^20^. Here, we employ both methods to assess the effects of spatial autocorrelation on our random forest models in predicting burn severity. To assess model scalability, we also train the model on half the fires in our training set to predict the other fires.

The model is applied to 8 major fires (totaling 917,919 acres or 3,715 km^2^) which burned across the state of New Mexico in 2021 and 2022 covering diverse ecoregions (forests, deserts, high plains), plant species, and topographies. These included high-severity fires such as Hermit’s Peak/Calf-Canyon, which was the largest recorded fire in state history and burned 341,735 acres, to low severity fires including Black Fire and Cerro Pelado. In addition, we also apply a generalized model trained in one region using half the fires, to predict burn occurrence in the other half of the fires to assess the feasibility of applying models outside the training area. This latter task is important for regions which may have not yet been burned, but where future wildfire occurrence is possible. Improving fire outlooks is critical, and models which can be generalized across states and regions and still produce fine-scale fire severity predictions are needed for management and pre-fire mitigation.

Our approach builds on past empirical studies^19–23,26^, and includes the use of fine-scale 70 m ECOSTRESS ET and ESI data, which can represent fuel type^37^, flammability (through the water stress metric) and amount (as represented by variations in ET). Other studies have indicated the importance of including information on fuels^30^, water stress^38^, in addition to topography and weather. Here we build on previous empirical studies, using information on weather, topography and fuels as represented by ECOSTRESS. Remote sensing data are needed at fine-scales given the highly complex and heterogeneous patterns of fuels and topography in remote terrain^38,39^, and the resulting burn severity complexity. We also build on previous studies^19,20,22^ to assess the role of spatial autocorrelation on our model prediction accuracy and scalability. We explore three main questions: (1) what are the most important predictors of burn severity across New Mexico?, (2) how does spatial autocorrelation influence model accuracy?, and (3) to what extent are burn severity predictions scalable across diverse landscape and ecosystem types? Although applied to New Mexico, the framework presented in this study has the potential to be extended to other states and regions.

## Methods

### Study region

**Fig. 1:**
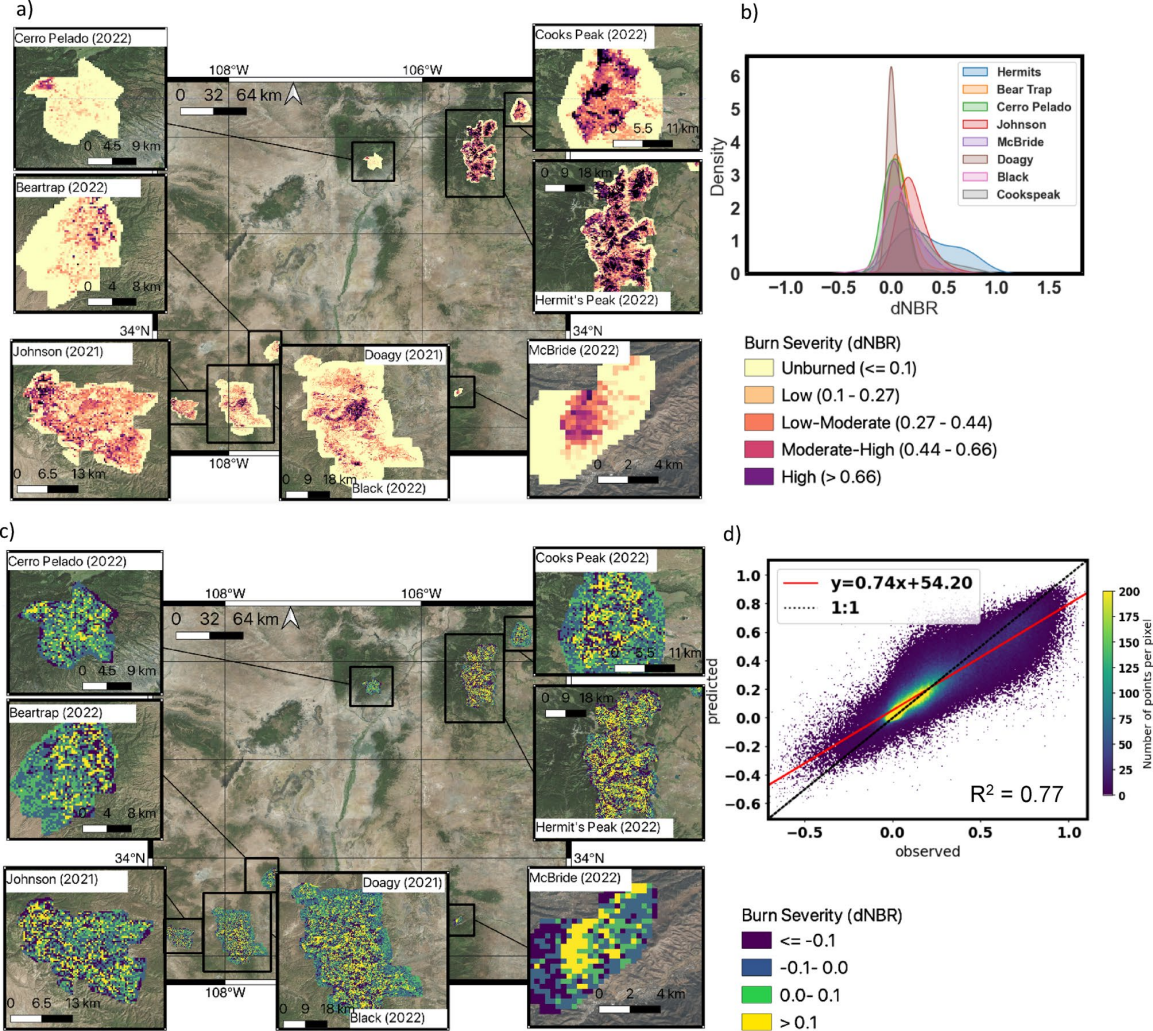
Burn severity predicted from weather, topography and fuels predictors agrees with observed patterns with a high degree of accuracy (R^2^ = 0.77; RMSE = 0.11) across diverse landscapes and conditions. (**a**) Observed wildfire burn severity (dNBR) (unitless from ~ − 0.5 to 1.5) for the 8 fires in New Mexico in 2021 and 2022 (Cooks Peak, Hermit’s Peak/Calf-Canyon, McBride, Black, Doagy, Johnson, Beartrap and Cerro Pelado). Colormap indicates burn severity classification ranging from unburned (< = 0.1), to high severity (> 0.66). (**b**) Kernel density estimator plot indicating the density distribution (y-axis) of values of burn severity (dNBR) (x-axis) for each of the 8 fires. (**c**) Observed minus predicted burn severity (dNBR) from Sentinel-2 for the 8 fires modeled fuel type and flammability (ECOSTRESS ET, ESI, topography) and weather (VPD and TMAX). Yellow shading indicates observed is greater than predicted, and purple indicates observed is less than predicted. (**d**) Density scatter plot of predicted (y-axis) against observed (x-axis) burn severity for the 8 fires modeled. Shading indicates density of points and is indicated by colormap. Red line is the linear fit of points. R-squared and equation are shown. Figure created using QGIS 3.18 (https://qgis.org/) software with the Bing Maps Satellite Imagery as a basemap (included with QGIS 3.18 software).

We focused on 8 large New Mexico wildfires which occurred in 2021 and 2022, including data from the 2022 record breaking year (Fig. 1). We look at fires from both 2022 and 2021 to have a larger training set than from a single year of fires, and to capture at least a full year of vegetation characteristics prior to the fire start from ECOSTRESS which began recording data in June 2018. The fires include: (1) the Hermit’s Peak/Calf-Canyon Fire, (2) the Black Fire, (3) the Cerro Pelado Fire, (4) the Bear Trap Fire, (5) the Cooks Peak Fire, (6) the McBride Fire, (7) the Johnson Fire, and (8) the Doagy Fire. Details on area burned, start and end dates are shown in Table 1. Fire information was obtained from Inciweb (https://inciweb.nwcg.gov), NM Fire Information (www.nmfireinfo.com), and NM Department of Homeland Security and Emergency Management (https://www.nmdhsem.org/2022-wildfires/).

**Table 1:** Dates and area burned for each fire.

	Fire	Start date	End date	Area burned (Acres)	Date of dNBR
1	Hermit’s Peak/Calf-Canyon	04–06-2022	08–21-2022	341,735	06–15-2022
2	Black	05–13-2022	07–27-2022	325,133	06–15-2022
3	Cerro Pelado	04–22-2022	06–15-2022	45,605	05–16-2022
4	Bear Trap	05–01-2022	06–05-2022	38,225	05–26-2022
5	Cooks Peak	04–17-2022	05–13-2022	59,359	05–12-2022
6	McBride	04–12-2022	05–07-2022	6159	04–18-2022
7	Johnson	05–20-2021	07–23-2021	88,918	06–19-2021
8	Doagy	05–14-2021	06–03-2021	12,785	06–03-2021

Fires arranged in descending order by year, and size (acres burned). We also include the date of the burn severity (dNBR) calculation post-fire containment.

### Burn severity

We use burn severity data produced by the Burned Area Emergency Response (BAER) team (data available online at: https://burnseverity.cr.usgs.gov/baer/). The BAER burn severity is produced immediately following containment of the fire. It therefore does not include vegetation mortality but captures a snapshot of conditions directly after the burn. It also does not include vegetation regrowth following a fire, compared to use of a burn severity estimate from one year-following the fire. Here, we decided to use the burn severity from immediately following the fire – but not the caveat that future vegetation die-off may be omitted. Another caveat is that uncertainty exists in satellite burn severity which may be coarser than fine-scale vegetation structure, leading to discrepancies with field-based in situ measurements^40^.

The burn severity data are calculated using differenced Normalized Burn Ratio (dNBR) using Sentinel-2 satellite imagery (for details see: Wasser (2018)). The dNBR is validated against in situ assessments of burn severity, to produce a final categorical soil burn classification product. In this study, we use the preliminary BAER products as we are interested in the continuous dNBR values, and also in the effects on the canopy and soils which is captured by dNBR. dNBR was not available from BAER for the Cooks Peak Fire. For this fire, we calculated dNBR using Harmonized Landsat Sentinel data (downloaded from https://appeears.earthdatacloud.nasa.gov) following^10^, and described in^22^ with the following:

1 $$NBR \, = \, \left( {NIR \, {-} \, SWIR} \right)/\left( {NIR + SWIR} \right)$$

where NIR is the near infrared, and SWIR is the shortwave infrared part of the electromagnetic spectrum. NBR can identify vegetation and soils which have been burned given that healthy vegetation reflects strongly in the NIR portion of the spectrum, and burned vegetation will reflect more strongly in the SWIR portion. When NBR is positive, this indicates productive vegetative areas (e.g. there is greater reflectance by healthy vegetation (NIR) compared to bare soils and rocks (SWIR))^14^. The dNBR was then calculated by taking the difference of the pre-fire NBR and post-fire NBR (which is obtained following total containment of the wildfires):

2 $$dNBR \, = \, pre - fire \, NBR \, {-} \, post - fire \, NBR$$

We use the following labels for dNBR (as in^10^, which are based on United States Geological Survey^14^): unburned (dNBR < 0.1), low severity (dNBR = 0.1 – 0.27), low-moderate severity (dNBR = 0.27–0.44), moderate-high severity (dNBR = 0.44 – 0.66), high severity (dNBR > 0.67). These thresholds are subject to uncertainty due to the timing (seasonality) of the imagery used, and if the post-fire scene is generally drier than the pre-fire scene which can elevate burn severity values^14^. For running the binary predictions using random forest classification, we focused on prediction of low and low-moderate fire severity using a threshold of dNBR = > 0.1 (Figure S1, right panel) and dNBR = > 0.27^10^ (Figure S1, left panel)). We then produced new masks for the dNBR data where all pixels with dNBR greater than 0.1 or 0.27 were marked as burned (Figure S1). The choice to use dNBR = > 0.27 arose from experimentation using different USGS thresholds (above), with the 0.27 (low-moderate) threshold yielding highest accuracy classifications.

### Hydrological variables

#### Plant water stress

We used the ECOSTRESS Level-3 daily Priestley-Taylor Jet Propulsion Laboratory (PT-JPL) ET (L3_ET_PT-JPL), and the Level-4 ESI (calculated as the ratio of ET to PET) , which have a spatial resolution of 70 m and a temporal revisit of 3–5 days^32^. ESI is a metric of how stressed a plant is by comparing their actual ET to PET and is a leading indicator of drought (Fisher et al., 2011). Level-3 ET is calculated using the PT-JPL algorithm, which converts potential ET (PET) (calculated based on a Priestly-Taylor (1972) formulation) to actual ET with ecophysiological constraints using the ECOSTRESS Level-2 Land Surface Temperature (LST) product and ancillary datasets^32^. ESI and WUE are Level-4 products, and use PT-JPL ET and ancillary data (including GPP in the case of WUE given it is calculated as the ratio of GPP to ET).

We obtained ET and ESI over the domain of each of the six fires for the entire year before the fire start date using the online AppEEARS tool (online at https://lpdaacsvc.cr.usgs.gov/appeears/). \ Scenes with more than 50% of points missing were also not used. In subsequent analyses, we used the time-averaged annual mean values, as well as the observations from the observation closest in time to the fire outbreak values for ET and ESI. The annual-time averaging was performed to capture longer-term conditions impacting plant stress, including the effects of previous season drought. The month-before the fire outbreak mean values include information as a snapshot of conditions directly before the start of the fire. The rationale for this, is to test the efficacy of running forecasts using current conditions to obtain information on whether a region is expected to have a severe burn. The values from the observation closest in time before the fire capture short term plant-stress caused by hot and dry weather conditions.

### Weather data

We use the meteorological variables VPD and maximum air temperature (TMAX) from gridMET, available at a spatial resolution of 4 km, and daily temporal resolution (https://www.climatologylab.org/gridmet.html). The data from gridMET are based on output from observations including the Parameter-elevation Regressions on Independent Slopes Model (PRISM) (https://www.prism.oregonstate.edu/), and output from the North American Land Data Assimilation System version 2 (NLDAS-2)^41^. The weather data are obtained for 1-week before the fire outbreak. Although the scale of the weather is relatively coarse (4 km), we expect the meteorological conditions to be more homogenous over larger scales compared with the fuels data as represented by ECOSTRESS.

### Topography and land cover

We used elevation data from the Shuttle Radar Topography Mission (SRTM) which is available at 30 m resolution and downloaded for the fires from the AppEEARS tool. We calculate angle and aspect using SRTM elevation. We also use MODIS Land Cover Type (MCD12Q1) Version 6 which provides land cover at yearly intervals at 500 m resolution. We downloaded land cover for each of the fires for the entire year before the fire from the AppEEARS tool.

### Data set re-gridding

All data sets are re-gridded to the same grid as the 70 m ECOSTRESS data. We used linear interpolation to regrid the finer resolution products which has been found to produce similar impacts to the final environmental datasets compared with other regridding methods^42^. Impacts from use of linear interpolation to underlying data increase as grid resolution increases. Burn severity (20 m) and topography (30 m) were regridded with linear interpolation to the ECOSTRESS resolution (70 m). Coarser resolution data (VPD and TMAX (4 km), and land cover (500 m)) were downscaled by creating a new finer resolution grid with the same values as the coarser grids. Although these data sets contain less spatial information than the finer data sets, it permitted a view of general conditions over the regions for the weather and land cover conditions.

Although the weather (TMAX and VPD) information is coarser (4 km) than the ECOSTRESS ET and ESI (70 m), and topography (30 m) and burn severity (20 m), we expect that the patterns of weather are more spatially coherent (compared to e.g. the varying ET and ESI which vary with land cover). The coarser meteorological data therefore can provide broader context compared to the detailed plant stress and topography information. Despite this, there is the potential of microclimates arising during and following the wildfires which could lead to regionally complex weather patterns^43^. Fine-scale meteorology is however still a big unknown, due to limited or unavailable data from a lack of dense weather stations. Improving fine-scale fire weather information is a key need identified by the scientific community^39^. Another question concerns the use of multiple scales in the random forest model prediction. The use of a finer grid overlaying the coarser grids (e.g. weather information) could lead to a reduction in prediction performance and loss of detail in more complex regions, as the covariates with coarser pixel resolutions may not include important landscape properties.

### Random forest modeling

We employ two different modeling strategies for the wildfire prediction. The first is prediction of continuous burn severity trained on all fires using random forest regression (4.8.1). Here the goal is to produce 70 m predictions of burn severity which we compare against observed burn severity. The second strategy is a binary prediction of burn occurrence as either burn (dNBR = > 0.1; burned with low severity or greater) or no-burn (dNBR < 0.1; unburned) using random forest classification (4.8.2). For the second strategy, we train the model on half the fires (4 fires) and predict the other half (4 fires). The motivation for this strategy is to assess the scalability of the model framework when predicting outside the training area.

#### Random forest regression

We use random forest regression to produce continuous predictions for fire severity^25^. Random forests have been used in previous studies for fire severity prediction, given their ability to model non-linear relationships between predictor and response variables^20,20,22^. We group all 8 fires together (all available data points ‘N’, equal to 1**,**970**,**746) and train the model using 50% of all available data points (N = 985,373) and test our model on the remaining 50% of the points from the training fires. The points were selected at random from the gridded data set containing all the fires. We report the *R*^*2*^ based on the independent validation (or test) set of data, which is equivalent to *R*^*2*^ in linear regression modelling. We also report variable importance, which is derived from random forest variable permutation. The variable importance is obtained by randomly permuting values for each of the predictors and assessing the resultant change in mean square error from the original out-of-bag variance explained for the model.

9 predictors were used to run the models: information on plant water stress (as represented by evapotranspiration (ET) which varies according to vegetation amount and ecosystem type^37^) and evaporative stress index (ESI) (which varies according to water stress and can indicate flammability) from 70 m ECOSTRESS data averaged for the full year before the fire as well as the observation closest in time before the fire outbreak. We also use topographic predictors including elevation, slope, and aspect derived from the Shuttle Radar Topography Mission (SRTM) 30 m digital elevation model, and also predictors representing weather conditions from gridMET (4 km maximum air temperature, and VPD) (Supplementary Information S5 – S13). These predictors also varied amongst fires, due to location, and varying land cover and topographic characteristics and are illustrated by the kernel density estimator plots (Supplementary Information S6 – S7). For each of the predictor datasets we regridded to the same grid as the 70m ECOSTRESS data (see Data set re-gridding).

#### Random forest classification

We use random forest classification to create categorical model for fire severity prediction. Our dependent variable is burned pixels (dNBR => 0.10 (low intensity burn) or dNBR => 0.27 (low-moderate intensity burn)). We use the burn severity maps to create categorical maps of burned pixels (assigned a value of 1), and unburned (assigned a value of 0). We choose to focus on prediction of burned pixels (dNBR => 0.1), and low-moderate severity (dNBR => 0.27), as the model performance for higher severity events was found to be insignificant (not shown).

The random forest classification model is trained on 4 of the fires: Black, Johnson, Cerro Pelado and Doagy. We train the model using 50% of all available data points ‘N’ (N = 541,253), and test our model on the remaining 50% of the points from the training fires. The model (trained on the 4 fires Black, Johnson, Cerro Pelado and Doagy), is then applied to the other 4 fires (Hermit’s Peak/Calf-Canyon, Cooks Peak, McBride and Beartrap). The predicted burned pixels are then compared against the observed severity. Initially we began with all predictors (ECOSTRESS, topography and weather) but found that prediction accuracy was very low when using the topography and weather predictors. We therefore present results for the model trained on ECOSTRESS variables only.

To assess model performance, we report the i) percent of pixels accurately classified (as in^28,29^), ii) percent of burned pixels accurately classified, iii) error of omission, and iv) error of commission. These are defined by the following:

3 $$Percent\, \, of\, \, pixels\, \, accurately\, \, classified \, = \, N_{correct} /N_{total,all}$$

4 $$Percent\, \, of\, \, burned\, \, pixels \, \,accurately\, \, classified \, = \, N_{correct,burned} /N_{total,burned}$$

5 $$Error \, \,of\, \, Omission \, = \, N_{false,negative} /N_{total,burned}$$

6 $$Error\, \, of\, \, Commission \, = \, N_{false,positive} /N_{total,burned}$$

where *N*_*correct*_ is correctly classified pixels (burn or no-burn), *N*_*total,all*_ is number of all pixels, *N*_*correct,burned*_ is number of correctly classified burned pixels, *N*_*total,burned*_ is total number of burned pixels, *N*_*false,negative*_ is number of pixels incorrectly classified as burned, *N*_*false,positive*_ is number of pixels incorrectly classified as burned.

In order to check for robustness of the results, we re-did the same analysis for 9 different groupings of the 4 model training fires and the 4 fires predicted (10 total groupings selected randomly) (Supplementary Information, Table S1). For each set of groupings we calculated the model accuracy (overall accuracy, burned pixel accuracy, error of omission, and error of commission).

#### Assessing how number of predictors influences prediction accuracy

We assessed how the number of predictors influences prediction accuracy (as reported by *R*^*2*^) by examining the effect of running the model (again at 70 m resolution) with 1 predictor (n = 1) and adding back in all predictors (n = 9). We did this in two ways: 1) starting with the most important predictor and adding back in each predictor in order of its importance (Supplementary Information, Figure S15), and 2) adding back in n number of predictors at random. For each of the 9 model runs we recorded the prediction *R*^*2*^ (Supplementary Information, Figure S15). We repeated this random method 25 times (25 different random variations of 1, 2, …n predictors). We then plotted the *R*^*2*^ as a function of the number of ‘n’ predictors used from these random combinations. We took the average of *R*^*2*^ for each of the 25 random combinations from n = 1 to n = 9 and plotted the average on the chart.

#### Spatial autocorrelation

##### Increased sample spacing: all fires

Spatial autocorrelation is prevalent in spatial ecological data sets, due to the greater relatedness of neighboring data points than would be expected from random points in space^44^. To assess the influence of spatial autocorrelation in the random forest models, we use the same methods to determine spatial autocorrelation described in^22^, which followed the methods in^20^. We assessed spatial autocorrelation by rerunning the model containing all 8 fires at increasing sample spacing: 280 m, 560 m, 840 m and 1120 m. In each case we used 50% of the points randomly selected from the re-gridded data set to train the model and kept the other 50% for testing. We reported R^2^, RMSE and top three most important variables. For consistency, we also re-ran the model for all fires at different sample spacings holding the number of points in the training set constant at N = 61,586. This number of points was selected as it represented the number of points used in training at the highest sample size of 1120 m.

##### Semi-variogram: Doagy fire

We used the semi-variogram technique to determine the distance at which spatial autocorrelation in the dataset decreases following methods described in^45,46^. The semi-variogram measures how dissimilar two observations are in relation to the distance between. For points that are nearby in space, the variance between the two points will be small, and increases as distance between the observations increases^46^. The variance between points increases up to a point as distance increases, until it tends to level off at a maximum value (this distance is known as the “sill”)^46^. After this distance, it can be assumed that there is no longer autocorrelation in the data. To calculate the semi-variogram we used the ‘scikit-gstat’ publicly available Python package^47^. We calculated the semi-variograms for distances up to 5000 m.

##### Principal coordinates of neighbor matrices: Doagy Fire

We used the Principal Coordinates of Neighbor Matrices (PCNM) method, which calculates variables representing spatial autocorrelation in the predictors. We adapted the ‘pcnm’ function from the vegan R package which is publicly available online (https://github.com/vegandevs/vegan/blob/master/man/pcnm.Rd)^48^ into Python. Given the high computational costs of running the pcnm function, as in^20,22^, for the analysis looking at all fires we use a single fire – the Doagy Fire – as a case study. We then calculated the first 3-PCNMs which we included as predictor variables in our random forest model. We re-ran the random forest model using the original 9 predictors for the Doagy Fire as well as the 3 PCNM predictors, and report the validation data *R*^*2*^ and variable importance (Supplementary Information, Table S2). We begin with no sample spacing (using points from the original gridded 70 m resolution data). We then increase the sample spacing to 280 m (regularly sampling every 4^th^ point), and 560 m (regularly sampling every 8th point). In each case we set aside 50% of the points for training the model and leave the other 50% for testing.

#### Predictor selection 

##### Random forest regression modeling

In our first experiment, we run the random forest classification using all 9 predictors (vegetation plant stress (ET and ESI annual mean and before fire), weather (VPD and TMAX) and topography (elevation, slope, aspect)) (Table 2). We then run additional model experiments systematically excluding different groups of predictors (vegetation plant stress, weather, and topography) to determine the most parsimonious set of predictors and report model performance. The resulting R^2^, and the individual sets of predictors used are recorded. For the random forest regression, we find the model results are highest (in terms of R^2^ when using all predictors). In addition, we also record the variable importance when running the random forest regression using all 9 predictors (Supplementary Information, Figure S14). We then run the model using n = 1 to n = 9 predictors, beginning with the most important predictor and adding back the next most important and so on (Supplementary Information, Figure S15).

**Table 2:** Predictor variables used in this study.

	Category	Variable	Mission/dataset	Spatial resolution	Temporal resolution
1	Vegetation plant stress	Evapotranspiration annual mean	ECOSTRESS PT_JPL L-3	70 m	3–5 days
2		Evapotranspiration before fire	ECOSTRESS PT_JPL L-3	70 m	3–5 days
3		Evaporative stress index – annual mean	ECOSTRESS PT_JPL L-4	70 m	3–5 days
4		Evaporative stress index – before fire	ECOSTRESS PT_JPL L-4	70 m	3–5 days
5	Weather	Vapor pressure deficit	gridMET	4 km	1 day
6		Air temperature	gridMET	4 km	1 day
7	Topography	Elevation	SRTM	30 m	
8		Slope	SRTM	30 m	
9		Aspect	SRTM	30 m	

We also re-run the random forest regression using random combinations of n = 1 to n = 9 predictors 25 times. Each time we record the predicted R^2^, and plot this as well as the mean R^2^ for each number of predictors (Supplementary Information, Figure S15).

##### Random forest classification modeling

For the random forest classification, we also ran systematic tests excluding groups of variables (not shown), and found the best modeled results for the prediction are returned when using only the vegetation plant stress predictors.

## Results

### Prediction of continuous burn severity over 8 different fires

We first investigated prediction of continuous burn severity at 70m for 8 large wildfires which occurred in New Mexico in 2021 and 2022, and which burned across a range of diverse ecoregions including deserts, forests, and high plains (Supplementary Information Figure S2). These ecoregions themselves contain a variety of plant species; grasslands, pinyon-juniper woodlands, forests, shrublands, riparian wetlands, alpine tundra, and ecotones which include a combination of multiple vegetation types^49^. These fires also burned across varying topographical settings and elevations (ranging from 870 m to over 3500 m) (Supplementary Information, Figures S3 – S5), with varying plant water stress and weather conditions (Supplementary Information, Figures S6 – S11). The 8 fires themselves burned at different severities; the Hermit’s Peak/Calf Canyon Fire where it burned was characterized by high severity (as represented by the immediate post-fire dNBR (equation defined in Methods Burn Severity)) (mean dNBR = 0.43, standard deviation = 0.23), while the Johnson and Cooks Peak Fires burned with moderate severities (dNBR = 0.25, standard deviation = 0.13; dNBR = 0.33, standard deviation = 0.18), and the Cerro Pelado and Black Fires burned with overall lower severities (dNBR = 0.16, standard deviation = 0.08; dNBR = 0.23, standard deviation = 0.13) (Figures 1a,b). We considered a categorical land cover predictor in our models, but this was removed because it did not improve model performance.

We trained a random forest regression model on burn severity (dNBR) from 50% of the points (N = 985,373) from the 8 fires using all 9 predictors (Table 2). The model produced a high accuracy prediction of burn severity across all 8 fires (R^2^ = 0.77; RMSE = 0.11) (Figure 1c,d). At lower severities and unburned pixels, the model was found to over-predict dNBR, while at higher severities the model underpredicted values of dNBR (Figure 1c,d) (consistent with previous results in Pascolini-Campbell et al. (2022)). For example, the regions of highest burn severity for Cooks Peak and McBride Fires were underestimated by the model compared to observations (difference in dNBR > 0.25 as indicated by yellow shading in Figure 1c). Using random forest model variable permutation for variable importance we found that VPD from the week before the fire was the most important variable followed by ESI nearest in time to the fire outbreak, TMAX from the week before the fire, elevation and annual mean ESI (Supplementary Information, Figure S14). The least important variables were found to be aspect and slope angle.

**Fig. 2:**
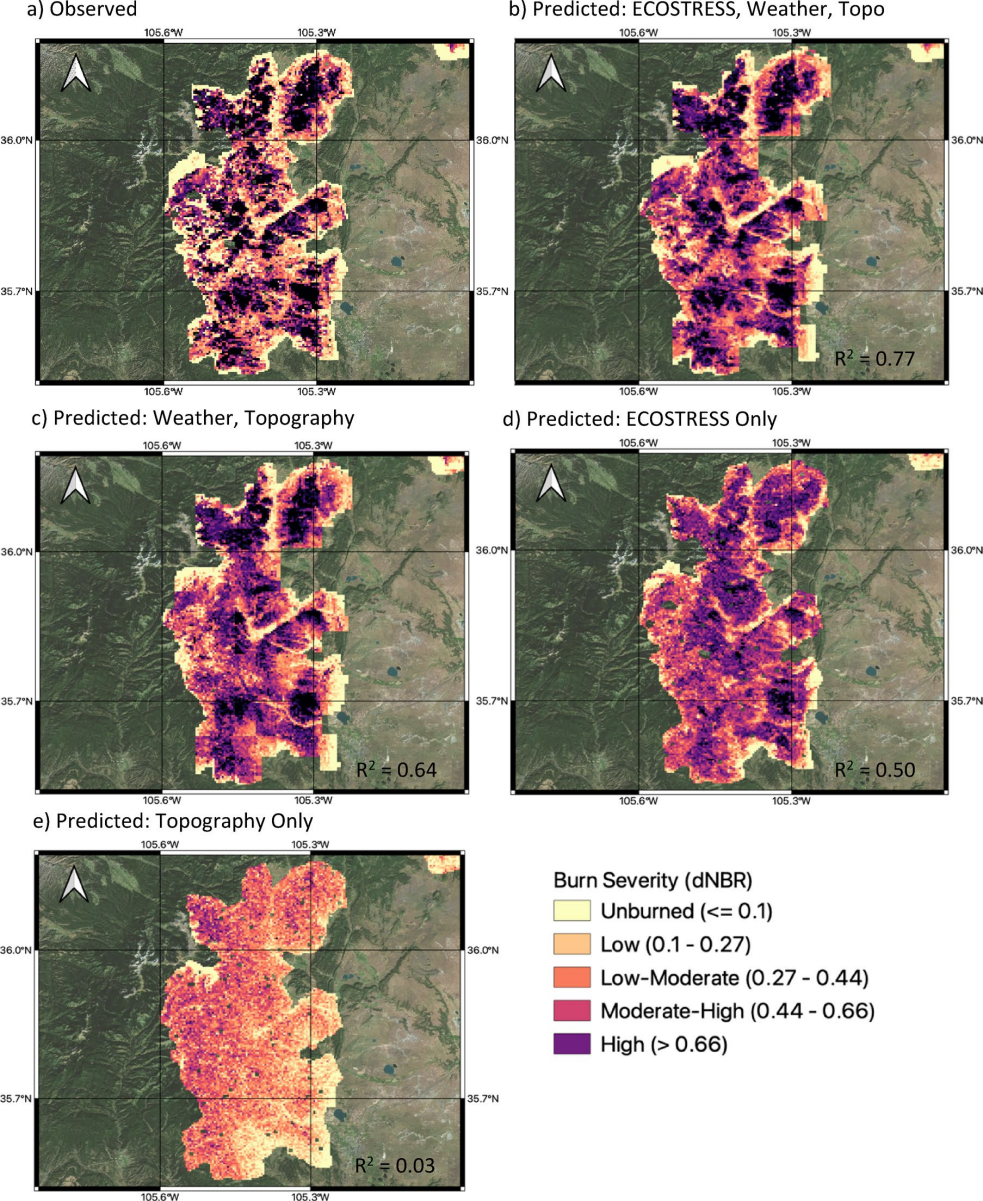
Effect of removing predictor variables on burn severity prediction accuracy shown for the Hermit’s Peak/Calf Canyon fire. (**a**) Observed Sentinel-2 burn severity (dNBR), (**b**) predicted dNBR using all predictors (ECOSTRESS, weather, topography), (**c**) predicted dNBR using weather and topography only, (**d**) predicted dNBR using ECOSTRESS only, (**e**) predicted dNBR using topography only. R-squared of the predicted versus observed dNBR is shown in bottom right corner of each plot. Figure created using QGIS 3.18 (https://qgis.org/) software with the Bing Maps Satellite Imagery as a basemap (included with QGIS 3.18 software).

We also assessed the model performance against observations for different sets of predictors, and present results zoomed in to the region of the Hermit’s Peak/Calf Canyon Fire (Figure 2, Table 3). The model was most accurate (across all 8 fires) when using all predictors (R^2^ = 0.77; RMSE = 0.11) (TMAX, VPD, elevation, slope, aspect, ECOSTRESS) (Figure 2b). With only weather and topography data (maximum air temperature (TMAX), VPD, elevation, slope, aspect) accuracy declines (R^2^ = 0.64; RMSE = 0.14) (Figure 2c). Running the model with ECOSTRESS (ET and ESI annual mean and nearest in time to fire) and topography (elevation, slope and aspect) returns an accuracy of R^2^ = 0.67 and RMSE = 0.13 (not shown). Running the model with only ECOSTRESS (ET and ESI annual mean and nearest in time to fire) returns an accuracy of R^2^ = 0.50 and RMSE = 0.17 (Figure 2d), running the model with only topography (elevation, slope and aspect) returns an accuracy of R^2^ = 0.03 and RMSE = 0.23 (Figure 2e). Running the model with only weather data yielded an accuracy of R^2^ = 0.46 and RMSE = 0.17, however it is not shown as results are spatially coarse due to the 4 km pixel resolution of the inputs.

**Table 3:** Predictors used in each random forest model run and corresponding R^2^ and RMSE.

Model run (number of predictors)	Predictors	R^2^	RMSE
All predictors (9)	ET annual mean ET before fire ESI annual mean ESI before fire VPD TMAX Elevation Slope Aspect	0.77	0.11
ECOSTRESS, topography (7)	ET annual mean ET before fire ESI annual mean ESI before fire Elevation Slope Aspect	0.67	0.13
Weather, topography (5)	VPD TMAX Elevation Slope Aspect	0.64	0.14
ECOSTRESS (4)	ET annual mean ET before fire ESI annual mean ESI before fire	0.50	0.17
Weather (2)	VPD TMAX	0.46	0.17
Topography (2)	Elevation Slope Aspect	0.03	0.23

Reducing the number of predictors drives down prediction accuracy. We investigated the effect of adding predictors in in order of variable importance, and at random (see *Methods 2.7.3*). We find that as expected prediction R^2^ increases as a function of the number of predictors included in the model (Supplementary Information Figure S14) (expected, due to more information available for model training). The R^2^ when adding back in most important predictors (orange line) increases faster than the when adding back in random combinations of ‘n’ predictors (green line) before converging when more (of the same) predictors are used.

### Spatial autocorrelation impact on model results

*Spatial autocorrelation assessment with semi-variogram:* We first assessed over what spatial distances we expect our results to be correlated. This was done by computing the semi-variogram– a geostatistics technique that determines at which distance spatial autocorrelation in the data are reduced^45,46^. We applied the technique to dNBR data from the Doagy Fire which serves as a good test case given its smaller size (12,785 Acres) and fewer data points (N = 27,783), allowing for this computationally intensive technique to be performed. We found that for the Doagy Fire the spatial autocorrelation decreases rapidly in the first 100 s of meters and begins to plateau at 1000 m to 2000 m (Supplementary Information, Figure S16). After 2000 m a maximum stable variance between data points is reached, indicating limited spatial autocorrelation after this distance.

*Sample spacing—all fires*: We assessed the impact of increasing the sample spacing on prediction accuracy (*Methods 2.7.4*). We found that model performance declined (as represented by R^2^ and RMSE) between the no spacing (70 m) test case, to the first spacing interval of 280 m (from R^2^ = 0.77 to R^2^ = 0.68; RMSE = 0.11 to RMSE = 0.13) (Table 4). For further increases in sample spacing to 560 m, 840 m and 1120 m there were further declines in model performance, though the declines appeared to level off. The reductions in model performance are expected due to both the reduced number of data points in the training set, as well as due to reduction in the effects of spatial autocorrelation. The most important variables were also stable, returning VPD, ESI nearest in time to fire outbreak, ESI year before fire, and elevation as the most important predictors.

**Table 4:** Summary of random forest model results for fires at various sample spacings, including R^2^, RMSE, and top three predictors.

Sample Spacing (m)	N_total_	N_train_	R^2^	RMSE	Variables	R^2^ (N_constant=_ 61,586)	RMSE (N_constant=_ 61,586)	Variables (N_constant=_ 61,586)
70	1,970,746	985,373	0.77	0.11	VPD ESI_nearest_ ESI_year_	0.62	0.15	ESI_nearest_ VPD Elevation
280	492,687	246,343	0.68	0.13	VPD ESI_nearest_ Elevation	0.61	0.15	ESI_nearest_ Elevation VPD
560	246,344	123,172	0.64	0.14	ESI_nearest_ VPD ESI_year_	0.60	0.15	ESI_nearest_ VPD ESI_year_
840	164,229	82,114	0.63	0.14	ESI_nearest_ VPD Elevation	0.61	0.15	VPD ESI_nearest_ Elevation
1120	123,172	61,586	0.60	0.15	ESI_nearest_ VPD ESI_year_	0.60	0.15	ESI_nearest_ VPD ESI_year_

Displays total points (N_total_) and training points (N_train_) for each case. Also includes results with a constant training set size (N_constant_ = 61,586).

For a more direct comparison between runs, we re-ran the random forest models across all fires using the same sample spacings (70 m up to 1120 m) but this time trained the models on the same number of points (using N = 61,586, which is 50% of the 1120 m sampling test case) to control for the effect of training data size on model performance (Table 4, right three columns). Unlike the prior analysis, we did not find any appreciable change between model performance (R^2^ and RMSE) for the different sample spacing intervals (Table 4, right three columns). The 70 m (no spacing) model had an accuracy of R^2^ = 0.62 and RMSE = 0.15, compared to the highest sample spacing (1120 m) R^2^ = 0.60 and RMSE = 0.15. This finding suggests that training set size apparently has a larger impact than spatial autocorrelation in driving model performance.

*PCNM—Doagy Fire:* We also assessed the importance of spatial autocorrelation in the data set by calculating the ‘principal coordinates of neighbor matrices’ (PCNM), following the methods of^20,21^ (*Methods, Sect. 2.8.4*). PCNM variables represent spatial autocorrelation – or the relatedness due to spatial proximity – in the data. As in^20,21^, we included the PCNMs as predictor variables in the random forest models using different grid spacing as above. We find that R^2^ is highest when no sample spacing is used (R^2^ = 0.64) and decreases at larger spacing intervals (R^2^ = 0.57, 280 m; R^2^ = 0.50, 560 m) (Supplementary Information, Table S2). In each case, we find the most important variable explained is ET from the year before the fire, indicating stability in the modeled results as in^19^. A spatial variable (PCNM) also is ranked as the next most important predictor returned.

### Scalability of random forest wildfire models

In this part of the study, we assess the scalability of the modeling framework by training the random forest model in one region (4 fires) to predict fire hazard in a different region (other 4 fires). The motivation for this part of the work is to determine to what extent these model approaches can be used to predict fire hazards in region that may have not yet burned in the past, or where observations of burn severity do not exist (and are therefore not included in the training set). We began using random forest regression, but predictions were very low accuracy (not shown). We then tested this new modeling strategy using classification based on a binary prediction of burned and unburned pixels.

**Fig. 3:**
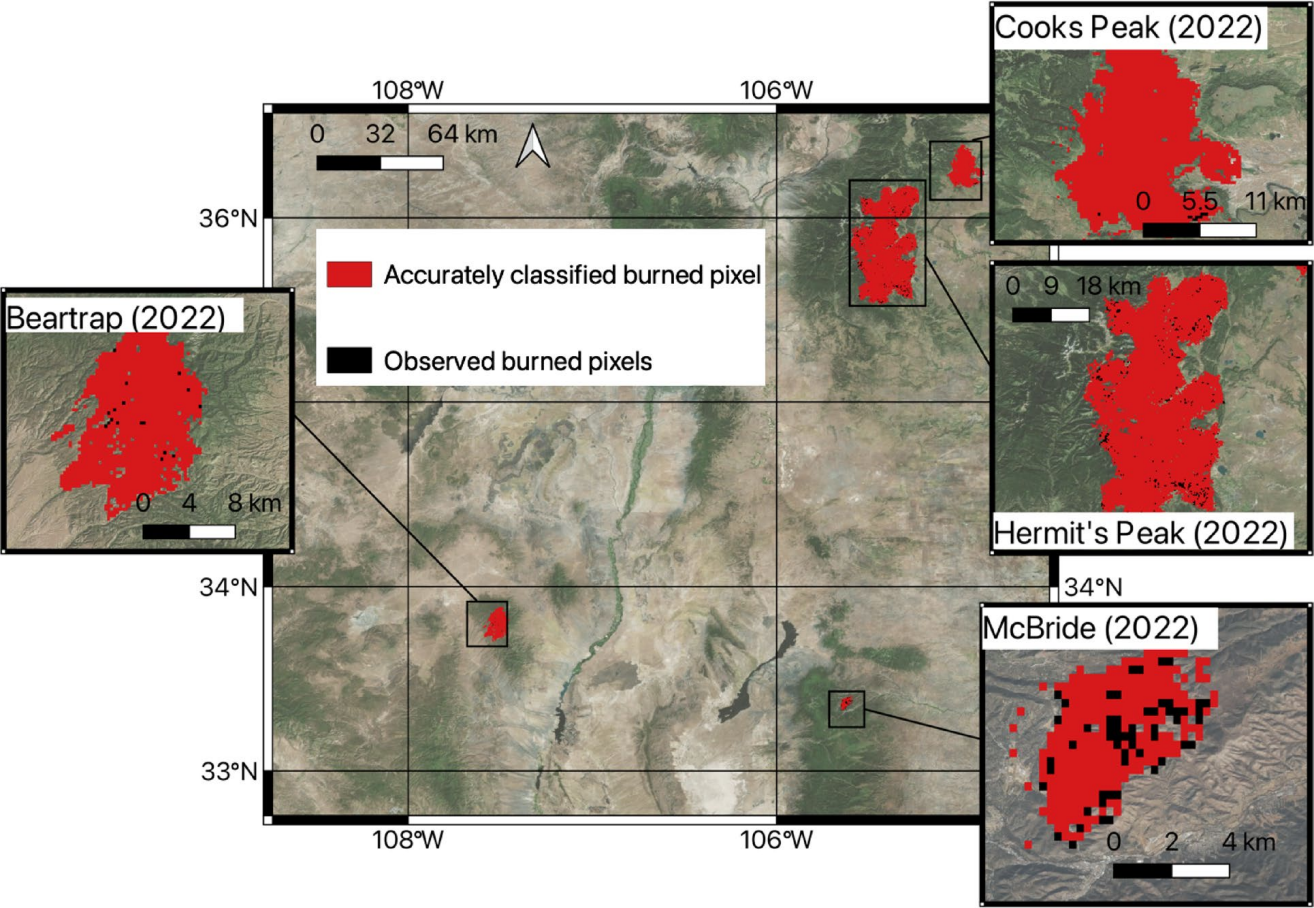
Burn occurrence (dNBR = > 0.1) predicted for half of the fires (Hermit’s Peak/Calf-Canyon, Beartrap, Cooks Peak, McBride from a random forest classification model trained on the other half (Cerro Pelado, Johnson, Doagy, Black). The red coloring indicates the pixels accurately classified (burned pixel (dNBR = > 0.1) predicted as burned, unburned pixel (dNBR < 0.1) predicted as unburned) by the random classification model, compared to the observed burned pixels (black) (dNBR = > 0.1). Figure created using QGIS 3.18 (https://qgis.org/) software with the Bing Maps Satellite Imagery as a basemap (included with QGIS 3.18 software).

We trained a random forest classification model on half of the fires (Doagy, Johnson, Cerro Pelado, Black) and predicted wildfire occurrence (binary classification of burn/no-burn) (as represented by dNBR = > 0.1 (burn) or dNBR < 0.1 (no-burn)) (see Methods Sect. 4.2, Fig. 4) for the other half of the fires (Hermit’s Peak/Calf-Canyon, Bear Trap, Cooks Peak, McBride) (Fig. 3). The most important set of predictors were found to be the 4 plant stress predictors: ECOSTRESS ET and ESI annual mean and closest in time to fire outbreak. Weather and topography were originally used as predictors but were found to produce low prediction accuracy and were then removed from the model. The random forest classification model (trained on burn (dNBR > = 0.1)/no-burn (dNBR < 0.1)) has an overall classification accuracy of 67.2% (for all pixels burned and non-burned) and classifies burned pixels with an accuracy of 85.5%. The percent of omission (i.e. pixels that burned which were not classified as burned) is 14.5%, and the error of commission (i.e. false positives) is 27.0%. In other words, the model is predicting fire hazard incorrectly for almost one-third of pixels identified as hazards in this case. The same method was applied but this time to predict low-moderate severity burned pixels and greater (dNBR = > 0.27), which yielded an overall accuracy of 54.6%, and classified burned pixels with an accuracy of 17%, with an error of omission of 83.0% and error of commission 56.8% (Supplementary Information, Figure S17). The choice to use dNBR = > 0.27 arose from experimentation using different thresholds, with the dNBR = > 0.10 (low) threshold yielding the highest accuracy classifications.

To check for robustness of the results, we re-did the same analysis for 9 different groupings of the 4 model training fires and the 4 fires predicted (10 total groupings selected randomly) (Supplementary Information, Table S1). Across the 10 groupings mean overall accuracy was 45.3% (st. dev. 33.4%), mean burned pixels accurately classified was 59.4 % (st. dev. 9.8%), mean error of omission was 54.7% (st. dev. 33.4%), and mean error of commission was 34.1% (st. dev. 15.0%).

## Discussion

Predictions of fire severity are needed for hazard monitoring, to inform management activities including setting low-severity prescribed burns, and as inputs to post-fire impact modeling such as debris flow and water quality. Given the highly heterogenous patterns of fuels, landscapes and resulting burn patterns, severity predictions are required at high (< 100 m) spatial resolution. Here we present an empirical approach to the wildfire modeling problem using 70 m ECOSTRESS ET and ESI data as metrics to represent fuel flammability and fuel type^37^. Topography also influences moisture, fuels, and burn severity, given that moisture and species composition tend to vary along different elevations and aspects^50^. Temperature and atmospheric demand are also positively correlated with fire intensity, with drier and warmer conditions leading to greater fire hazard^51^. The proliferation of remote sensing data offers a path forward by providing heterogeneous and dynamic measurements of plant water stress^52^, topography, weather and fire relevant quantities.

In general, we found the random forest regression model performed well across the 8 fires in New Mexico, which burned with diverse regimes (low to high burn severity), and in different topographic and ecological settings. We also note that our model over-predicted regions of low burn severity and under-predicted regions of high burn severity. This is a potential limitation to employing the methodology for risk preparedness and could mean that insufficient resources are deployed to high severity regions. The over-prediction of low-severity burning could also lead to inappropriate allocation of preventative fire-clearing activities. Potential ways to address these limitations are explored below.

We found including high resolution ECOSTRESS with weather and topography data improves the overall predictability of burn severity using random forest modeling. Part of the region in New Mexico that burned had evergreen forests, characterized by deep-rooted systems. In deeper rooted systems, there may be a time lag between moisture supply (precipitation) and ET/ESI^53^, which is sourced from deeper rootzone soil moisture stores. Observations of ET/ESI will still be able to capture vegetation water stress and dryness of these deep-rooted systems. ESI is also somewhat novel in fire analysis and is powerful as a direct high-resolution indicator of plant stress/dryness across different ecosystems. ET and ESI also both vary depending on the underlying vegetation cover; for example, well-watered grass exhibits maximal evapotranspiration, whereas well-watered forest may actually have less ET than grass due to aerodynamic resistances and branch shading. Differences between ET and ESI signals can also therefore serve as proxies of vegetation type^37^. Our findings indicate the potential of applying ET and ESI to wildfire prediction. We also found that weather (VPD and air temperature) played an important role in prediction, whereas topographical variables (especially slope and aspect) were less important, suggesting the spatial patterns of severity can be sufficiently characterized using plant stress and weather alone. It also suggests the plant stress metrics may be adequate for resolving the topographical influences on fuel moisture due to elevational and aspect gradients. 

The prediction accuracy of wildfire models is also driven by inherent spatial autocorrelation of the environmental data, as predictors share significant spatial variance with the target variable (dNBR)^19^. This arises from shared spatial patterns across variables like plant stress, weather, and topography which interact to produce the observed patterns of burn severity (which itself is also spatially autocorrelated)^20^. Understanding the impact of spatial autocorrelation on wildfire models is essential for producing models that can meaningfully capture the complex relationship between fuels, landscapes and burn severity, improve generalization, and avoid model overfitting. We found an apparent greater impact of training data set size versus spatial autocorrelation in impacting model accuracy. This suggests that the random forest model can capture complex fine-scale behavior resulting in burn severity patterns. It also points to the importance of large training sets for improving accuracy of predictions across diverse landscapes. Further work is needed to determine whether these results hold true for different ecosystems, regions and time periods.

We also assessed the scalability of the wildfire prediction models. We found random forest regression models to predict continuous burn severity performed poorly when scaled to other regions (i.e. for predicting regions not included in training). This could be due to the wildfires considered having taken place in different ecosystems, landscapes and burning with different intensities. Random forest classification was instead used for a binary prediction of burned pixels and achieved greater results with an overall accuracy of 67.2%, burned pixel accuracy of 85.5% of burned pixels, and false positive rate at 27%. While use of more predictors often improves model accuracy, we found that use of all predictors (ECOSTRESS, topography and weather) resulted in a low overall accuracy. Instead using only ECOSTRESS variables led to the highest accuracy results when scaling to other regions. One implication of our finding is the importance of including fuels information (as represented here by ET and ESI), which was found to improve the scalability of our models. We note the false positive rate indicates that approximately one-third of the pixels identified as a fire hazard were classified incorrectly by the model. This presents a potential barrier for adapting this framework for prescribed burns, and further work is needed for scaling the predictions to regions outside the training set.

Future analyses could involve a more extensive training set over a range of climatic conditions to address the limitations with scaling. The present study only considered the years 2021 and 2022. In addition, other limitations in the current study could also contribute to the poor scalability of continuous burn severity in other regions. These limitations include the use of data sets with different resolution, in particular coarse meteorology (temperature and vapor pressure deficit), which will not be able to resolve microclimates existing in the landscape, but which could influence wildfire activity^43^. Higher resolution weather is currently limited by a lack of in situ monitoring but has been identified as a major need for the wildfire science and applications community^39^. In addition, the ECOSTRESS ET and ESI data are capturing surface water use of vegetation and do not consider other fuel related variables such as fuel water content and canopy structure. ECOSTRESS ET also does not specifically characterize rooting depth which can impact vegetation water storage, though ECOSTRESS has been found to be correlated with water usage at depth, when ECOSTRESS LST was coupled with soil moisture profiles^54^. Future investigations could also include other fuels relevant remotely sensed variables such as fuel moisture^55^ and canopy height^56^, and high resolution information on land cover type.

## Conclusion

Enabling observational techniques from remote sensing platforms like ECOSTRESS can provide fire danger forecasts that can be used in hazard assessment, planning prescribed burns and informing post-fire impacts. The results indicate promising results for applied science to generate fine-scale predictions of burn severity and burn occurrence across complex landscapes using high-spatial resolution, and frequently observed data using remote sensing (e.g., from ECOSTRESS). Further, anticipated missions such as NASA’s Surface Biology and Geology (S.B.G) will provide a continuation of the ECOSTRESS ET and ESI measurements at 60 m every 3 days. This study therefore also demonstrates future capabilities in wildfire science and applications.

## Electronic supplementary material

Below is the link to the electronic supplementary material.

Supplementary Material 1.

## Data Availability

ECOSTRESS PT-JPL daily ET, ESI and WUE, MODIS Land Cover Type (MCD12Q1), SRTM660 30 topography data, and Harmonized Landsat Sentinel (HLS) data are available at: https://lpdaacsvc.cr.usgs.gov/appeears/ VPD and TMAX are available at: https://www.climatologylab.org/gridmet.html BAER preliminary burn severity dNBR is available at: https://burnseverity.cr.usgs.gov/baer/.
