# Supplementary material for: Assessment of spatial autocorrelation and scalability in fine-scale wildfire random forest prediction models
Source: Sci Rep. 2025 Jul 1;15:21504. doi: 10.1038/s41598-025-06814-z (PMC12215841; doi:10.1038/s41598-025-06814-z)
Supplement: Supplementary file 1 — Supplementary Material 1. [file 41598_2025_6814_MOESM1_ESM.docx]

**Figure S1** **Burn/no-burn binary mask example**. *Pixels of the Hermit’s Peak/Calf-Canyon Fire which burned with a severity of dNBR => 0.27 (low-to-moderate severity and greater) (left panel), or a severity of dNBR => 0.10 (low severity) (right panel). Figure created using QGIS 3.18 (https://qgis.org/) software with the Bing Maps Satellite Imagery as a basemap (included with QGIS 3.18 software).*

**Figure S2** MODIS land cover (LC-TYPE2; University of Maryland classification) for each of the 8 fires. Figure created using QGIS 3.18 (https://qgis.org/) software with the Bing Maps Satellite Imagery as a basemap (included with QGIS 3.18 software).

**Figure S3** Elevation (meters) derived from the Shuttle Radar Topography Mission (SRTM) Digital Elevation Model (DEM) at 30 m resolution. Figure created using QGIS 3.18 (https://qgis.org/) software with the Bing Maps Satellite Imagery as a basemap (included with QGIS 3.18 software).

**Figure S4** Slope angle (degrees) derived from the Shuttle Radar Topography Mission (SRTM) Digital Elevation Model (DEM) at 30 m resolution. Figure created using QGIS 3.18 (https://qgis.org/) software with the Bing Maps Satellite Imagery as a basemap (included with QGIS 3.18 software).

**Figure S5** Aspect (from 0 to 360 degrees where 0 represents north) derived from the Shuttle Radar Topography Mission (SRTM) Digital Elevation Model (DEM) at 30 m resolution. Figure created using QGIS 3.18 (https://qgis.org/) software with the Bing Maps Satellite Imagery as a basemap (included with QGIS 3.18 software).

**Figure S6** Kernel density estimator plots indicating the distribution of values of a) elevation (meters), b) slope (degrees), and c) aspect (degrees) for each of the 8 fires.

**Figure S7** Kernel density estimator plots indicating the distribution of values of a) ECOSTRESS evaporative stress index (ESI) (ratio 0 – 1) annual mean (for the year before fire outbreak), b) ESI before the fire, c) evapotranspiration (ET) (W/m2) annual mean (for the year before fire outbreak), d) ET (W/m2) before the fire , e) vapor pressure deficit (VPD) (kPa) from one week before fire outbreak, f) maximum air temperature (TMAX) (degrees Kelvin) for one week before fire outbreak for all 8 fires.

**Figure S8** Observed annual mean evapotranspiration (ET) (W/m2) averaged over the year leading up to fire outbreak from the ECOSTRESS PT-JPL L-3 ET product for each of the 8 fires. Figure created using QGIS 3.18 (https://qgis.org/) software with the Bing Maps Satellite Imagery as a basemap (included with QGIS 3.18 software).

**Figure S9** Observed evapotranspiration (ET) (W/m2) nearest in time to fire outbreak from the ECOSTRESS PT-JPL L-3 ET product for each of the 8 fires. Figure created using QGIS 3.18 (https://qgis.org/) software with the Bing Maps Satellite Imagery as a basemap (included with QGIS 3.18 software).

**Figure S10:** Observed annual mean evaporative stress index (ESI) (ratio 0 - 1) averaged over the year leading up to fire outbreak from the ECOSTRESS PT-JPL L-4 ESI product for each of the 8 fires. Figure created using QGIS 3.18 (https://qgis.org/) software with the Bing Maps Satellite Imagery as a basemap (included with QGIS 3.18 software).

**Figure S11:** Observed evaporative stress index (ESI) (ratio 0 - 1) nearest in time to fire outbreak from the ECOSTRESS PT-JPL L-4 ESI product for each of the 8 fires. Figure created using QGIS 3.18 (https://qgis.org/) software with the Bing Maps Satellite Imagery as a basemap (included with QGIS 3.18 software).

**Figure S12:** Vapor pressure deficit (VPD) (kPa) from one week before fire outbreak for each of the 8 fires. Figure created using QGIS 3.18 (https://qgis.org/) software with the Bing Maps Satellite Imagery as a basemap (included with QGIS 3.18 software).

**Figure S13:** Maximum air temperature (TMAX) (degrees Kelvin) for one week before fire outbreak for each of the 8 fires. Figure created using QGIS 3.18 (https://qgis.org/) software with the Bing Maps Satellite Imagery as a basemap (included with QGIS 3.18 software).

**Figure S12:** Maximum air temperature (TMAX) (degrees Kelvin) for one week before fire outbreak for each of the 8 fires.

**S**

**S14** Predicted burn severity (dNBR) for all fires at 70 m sample spacing, and variable importances from permutation. Figure created using QGIS 3.18 (https://qgis.org/) software with the Bing Maps Satellite Imagery as a basemap (included with QGIS 3.18 software).

**S15** Effect of the number of predictors (x-axis) on random forest R^2^ (y-axis). Random forest model is run from n=1 predictor to n=9 (all) predictors, starting with the most important predictor and adding back in order of importance (orange line). The random forest model is also run using random combinations of n=1 to n=9 predictors, and this is repeated 25 times (blue dots), and also the average is plotted (gray line).

Effect of Number of Predictors on R^2

|  | Most to Least Important Predictor |
| --- | --- |
| 1 | 0.37 |
| 2 | 0.3 |
| 3 | 0.58 |
| 4 | 0.66 |
| 5 | 0.7 |
| 6 | 0.73 |
| 7 | 0.76 |
| 8 | 0.77 |
| 9 | 0.78 |

|  | Random Combination Predictor |
| --- | --- |
| 8 | 0.771203 |
| 7 | 0.744294 |
| 6 | 0.670232 |
| 5 | 0.65024 |
| 4 | 0.583053 |
| 3 | 0.483811 |
| 2 | 0.258736 |
| 1 | 0.37152 |
| 8 | 0.764785 |
| 7 | 0.722735 |
| 6 | 0.686945 |
| 5 | 0.700786 |
| 4 | 0.570895 |
| 3 | 0.302644 |
| 2 | 0.231667 |
| 1 | 0.081797 |
| 8 | 0.750086 |
| 7 | 0.758231 |
| 6 | 0.661108 |
| 5 | 0.515053 |
| 4 | 0.534839 |
| 3 | 0.456158 |
| 2 | 0.0719428 |
| 1 | 0.370259 |
| 8 | 0.72858 |
| 7 | 0.742679 |
| 6 | 0.608351 |
| 5 | 0.578685 |
| 4 | 0.436546 |
| 3 | 0.501774 |
| 2 | 0.188494 |
| 1 | -0.0238123 |
| 8 | 0.765005 |
| 7 | 0.743708 |
| 6 | 0.745264 |
| 5 | 0.618989 |
| 4 | 0.535766 |
| 3 | 0.333954 |
| 2 | 0.24294 |
| 1 | 0.369629 |
| 8 | 0.766728 |
| 7 | 0.743795 |
| 6 | 0.726418 |
| 5 | 0.71223 |
| 4 | 0.391645 |
| 3 | 0.499939 |
| 2 | 0.480255 |
| 1 | 0.071137 |
| 8 | 0.729304 |
| 7 | 0.761189 |
| 6 | 0.744032 |
| 5 | 0.67677 |
| 4 | 0.58066 |
| 3 | 0.295112 |
| 2 | 0.262928 |
| 1 | -0.0226045 |
| 8 | 0.760382 |
| 7 | 0.745576 |
| 6 | 0.688989 |
| 5 | 0.68907 |
| 4 | 0.507084 |
| 3 | 0.30359 |
| 2 | 0.188478 |
| 1 | -0.0226603 |
| 8 | 0.747586 |
| 7 | 0.734795 |
| 6 | 0.696132 |
| 5 | 0.682251 |
| 4 | 0.391296 |
| 3 | 0.498733 |
| 2 | 0.349874 |
| 1 | 0.372136 |
| 8 | 0.749907 |
| 7 | 0.727158 |
| 6 | 0.684252 |
| 5 | 0.595335 |
| 4 | 0.360334 |
| 3 | 0.312928 |
| 2 | 0.265266 |
| 1 | -0.0233707 |
| 8 | 0.727912 |
| 7 | 0.743052 |
| 6 | 0.607799 |
| 5 | 0.577867 |
| 4 | 0.584895 |
| 3 | 0.345424 |
| 2 | 0.256528 |
| 1 | -0.0422745 |
| 8 | 0.765416 |
| 7 | 0.743708 |
| 6 | 0.734109 |
| 5 | 0.714114 |
| 4 | 0.547835 |
| 3 | 0.440551 |
| 2 | 0.218459 |
| 1 | -0.0851988 |
| 8 | 0.765039 |
| 7 | 0.745687 |
| 6 | 0.647758 |
| 5 | 0.607004 |
| 4 | 0.65043 |
| 3 | 0.437388 |
| 2 | 0.320588 |
| 1 | -0.177168 |
| 8 | 0.749875 |
| 7 | 0.74188 |
| 6 | 0.683865 |
| 5 | 0.584181 |
| 4 | 0.574839 |
| 3 | 0.439212 |
| 2 | 0.206402 |
| 1 | -0.0411588 |
| 8 | 0.764953 |
| 7 | 0.711472 |
| 6 | 0.570479 |
| 5 | 0.651704 |
| 4 | 0.50568 |
| 3 | 0.374039 |
| 2 | 0.244501 |
| 1 | 0.0757025 |
| 8 | 0.755718 |
| 7 | 0.737126 |
| 6 | 0.701811 |
| 5 | 0.651096 |
| 4 | 0.50082 |
| 3 | 0.521644 |
| 2 | 0.171838 |
| 1 | -0.178341 |
| 8 | 0.771804 |
| 7 | 0.762882 |
| 6 | 0.69303 |
| 5 | 0.653754 |
| 4 | 0.42532 |
| 3 | 0.457252 |
| 2 | 0.165156 |
| 1 | -0.175582 |
| 8 | 0.757939 |
| 7 | 0.763085 |
| 6 | 0.650021 |
| 5 | 0.502554 |
| 4 | 0.429397 |
| 3 | 0.468796 |
| 2 | 0.198351 |
| 1 | 0.37401 |
| 8 | 0.729878 |
| 7 | 0.733369 |
| 6 | 0.570968 |
| 5 | 0.679704 |
| 4 | 0.499624 |
| 3 | 0.439638 |
| 2 | 0.263411 |
| 1 | -0.00709301 |
| 8 | 0.756325 |
| 7 | 0.672976 |
| 6 | 0.740906 |
| 5 | 0.644306 |
| 4 | 0.536802 |
| 3 | 0.313849 |
| 2 | 0.191188 |
| 1 | 0.0704934 |
| 8 | 0.757073 |
| 7 | 0.705818 |
| 6 | 0.728718 |
| 5 | 0.704816 |
| 4 | 0.459237 |
| 3 | 0.606949 |
| 2 | 0.188147 |
| 1 | 0.37066 |
| 8 | 0.746978 |
| 7 | 0.689452 |
| 6 | 0.649238 |
| 5 | 0.638683 |
| 4 | 0.620761 |
| 3 | 0.426344 |
| 2 | 0.220931 |
| 1 | 0.0815472 |
| 8 | 0.747596 |
| 7 | 0.748452 |
| 6 | 0.728165 |
| 5 | 0.691572 |
| 4 | 0.686608 |
| 3 | 0.347418 |
| 2 | 0.206214 |
| 1 | -0.0226138 |
| 8 | 0.765001 |
| 7 | 0.706896 |
| 6 | 0.64482 |
| 5 | 0.574401 |
| 4 | 0.61797 |
| 3 | 0.38746 |
| 2 | 0.481978 |
| 1 | 0.0828403 |
| 8 | 0.771784 |
| 7 | 0.6898 |
| 6 | 0.659907 |
| 5 | 0.594796 |
| 4 | 0.611612 |
| 3 | 0.401112 |
| 2 | 0.319964 |
| 1 | -0.0219197 |

|  | Random Combination Average |
| --- | --- |
| 9 | 0.77 |
| 8 | 0.754674 |
| 7 | 0.732793 |
| 6 | 0.676933 |
| 5 | 0.635598 |
| 4 | 0.525758 |
| 3 | 0.415829 |
| 2 | 0.247769 |
| 1 | 0.0739174 |

**S16** Semi-variogram computed for differenced normalized burn ration (dNBR) for the Doagy Fire. The y-axis indicates the semivariance between points and the x-axis indicates the lags (distance in meters) between points. The top panel shows the number of points (N) included in the analysis up to a maximum distance of 5000 m.

**S17 *Burn occurrence (dNBR => 0.10) predicted for half of the fires (Hermit’s Peak/Calf-Canyon, Beartrap, Cooks Peak, McBride from a random forest classification model trained on the other half (Cerro Pelado, Johnson, Doagy, Black).***  *The red coloring indicates the pixels accurately classified (burned pixel (dNBR => 0.10) predicted as burned, unburned pixel (dNBR < 0.10) predicted as unburned) by the random classification model, compared to the observed burned pixels (black) (dNBR => 0.10). Figure created using QGIS 3.18 (https://qgis.org/) software with the Bing Maps Satellite Imagery as a basemap (included with QGIS 3.18 software).*

| Doagy  Johnson  Cerro  Black | McBride  Beartrap  Cookspeak  Hermits | Percent Accurately Classified as Burn: 85.5  Percent Accurately Classified: 67.2  Percent Omission: 14.5  False Positive / Error of Commission: 27.0 |
| --- | --- | --- |
|  |  |  |
| Doagy  Johnson  Cerro  Mcbride | Beartrap  Cookspeak  Hermits  Black | Percent Accurately Classified as Burn: 46.1  Percent Accurately Classified: 59.0  Percent Omission: 53.9  False Positive / Error of Commission: 24.2 |
|  |  |  |
| Doagy  Johnson  McBride  Beartrap | Cookspeak  Hermits  Black  Cerro | Percent Accurately Classified as Burn: 62.5  Percent Accurately Classified: 64.7  Percent Omission: 37.5  False Positive / Error of Commission: 25.8 |
|  |  |  |
| Doagy  Mcbride  Beartrap  Cookspeak | Hermits  Black  Cerro  Johnson | Percent Accurately Classified as Burn: 30.0  Percent Accurately Classified: 51.7  Percent Omission: 70.0  False Positive / Error of Commission: 19.2 |
|  |  |  |
| Johnson  Cerro  Black  Mcbride | Hermits  Doagy  Beartrap  Cookspeak | Percent Accurately Classified as Burn: 90.6  Percent Accurately Classified: 74.6  Percent Omission: 9.42  False Positive / Error of Commission: 22.7 |
|  |  |  |
| Johnson  Cerro  Black  Beartrap | Hermit  Doagy  McBride  Cookspeak | Percent Accurately Classified as Burn: 83.0  Percent Accurately Classified: 69.1  Percent Omission: 17.0  False Positive / Error of Commission: 23.2 |
|  |  |  |
| Johnson  Cerro  Doagy  Hermits | Mcbride  Cookspeak  Black  Beartrap | Percent Accurately Classified as Burn: 23.7  Percent Accurately Classified: 51.2  Percent Omission: 76.3  False Positive / Error of Commission: 56.6 |
|  |  |  |
| Johnson  Hermits  Doagy  Beartrap | McBride  Cookspeak  Cerro  Black | Percent Accurately Classified as Burn: 25.9  Percent Accurately Classified: 58.4  Percent Omission: 74.1  False Positive / Error of Commission: 41.8 |
|  |  |  |
| Johnson  Hermits  McBride  Cookspeak | Black  Cerro  Doagy  Beartrap | Percent Accurately Classified as Burn: 2.33  Percent Accurately Classified: 57.1  Percent Omission: 97.7  False Positive / Error of Commission: 61.6 |
|  |  |  |
| Black  Hermits  Cookspeak  Cerro | Doagy  Mcbride  Johson  Beartrap | Percent Accurately Classified as Burn: 3.37  Percent Accurately Classified: 41.4  Percent Omission: 96.6  False Positive / Error of Commission: 38.4 |

**Table S1 Prediction of burned pixels (dNBR => 0.1) for groupings of four fires.**

| Grid Spacing (m) | R^2^ | Top 3 Most Important Predictors |
| --- | --- | --- |
| 70 (no spacing) | 0.64 | ET year  PCNM2  PCNM1 |
| 280 | 0.57 | ET year  PCNM2  ET before fire |
| 560 | 0.50 | ET year  PCNM2  Aspect |

**Table S2 Effect of sample grid spacing on R^2^ and variable importance for the Doagy Fire using all 9 predictors and PCNMs 1 – 3 spatial autocorrelation predictors**
